# Remarkable Longevity of Herbarium-Derived Seeds of the Rare and Threatened Annual Legume *Astragalus contortuplicatus* L.: Germination After More Than 142 Years of Dry Storage

**DOI:** 10.3390/plants15142156

**Published:** 2026-07-13

**Authors:** Attila Molnár V., Henrietta Bak, Timea Nagy, Szabolcs Kis, Réka Fekete, Attila Takács

**Affiliations:** 1 HUN-REN–UD Conservation Biology Research Group, Department of Botany, University of Debrecen, Egyetem sq. 1, H-4032 Debrecen, Hungarykis.szabi17@gmail.com (S.K.); limodorum.abortivum@gmail.com (A.T.); 2 Juhász-Nagy Pál Doctoral School, University of Debrecen, Egyetem sq. 1, H-4032 Debrecen, Hungary; 3 Department of Pharmacognosy, Faculty of Pharmacy, University of Debrecen, Rex Ferenc u. 1, H-4002 Debrecen, Hungary; 4 Botanical Garden, University of Debrecen, Egyetem sq. 1, H-4032 Debrecen, Hungary

**Keywords:** ex situ conservation, Fabaceae, germination dynamics, long-term storage, physical dormancy, seed ageing, seed bank ecology, seed longevity

## Abstract

Seed longevity plays a crucial role in the persistence of plant populations, particularly in short-lived species inhabiting unpredictable environments. However, empirical data on long-term seed viability remain scarce, especially for rare and poorly known taxa. *Astragalus contortuplicatus* is a threatened annual species of periodically flooded habitats, characterized by sporadic occurrence and limited ecological knowledge; based on its annual life cycle and the temporal unpredictability of its habitat, its long-term population persistence is likely facilitated by persistent seed banks. In a previous study, we demonstrated that seeds of this species stored in herbarium collections can retain the ability to germinate for more than a century. Here, we reassess seed germination capacity after an additional 11.5–12 years of dry storage, using the same germination protocol, and analyse temporal changes based on repeated measurements of the same herbarium specimens. Germination data from 2014 and 2026 were evaluated using binomial models, and the relationship between seed age and germination capacity was examined using linear and non-linear approaches. Paired analyses revealed a significant overall decline in germination probability over time, although responses varied among specimens. Despite this decline, seeds from the oldest available sample (collected in 1883) still retained the ability to germinate, with 20% germination after approximately 142.5 years of storage. Across the full dataset, seed age had a significant negative effect on germination, and non-linear models provided a better fit than simple linear regression, indicating that the loss of germination capacity does not follow a strictly linear pattern. Our results confirm the exceptional longevity of seeds in this rare species while also demonstrating that germination capacity declines over time under continued dry storage. These findings are consistent with the ecological expectation that long-lived, dormant seeds may contribute to population persistence in unpredictable habitats, while underlining the potential of herbarium collections as valuable resources for the conservation and possible restoration of threatened plant species. Notably, seeds in the oldest sample retained the ability to germinate, emphasizing the exceptional longevity of seeds in this species.

## 1. Introduction

Long-term seed viability is a key ecological trait determining the persistence of plant populations in temporally variable and unpredictable environments. Persistent seed banks buffer fluctuations in recruitment and enable species to survive periods unsuitable for establishment [1,2]. This function is particularly important for short-lived annual species inhabiting dynamic habitats such as river floodplains, where environmental conditions may vary strongly among years.

Seeds of many *Fabaceae* species are characterized by physical dormancy caused by a water-impermeable seed coat, which can substantially delay germination and contribute to long-term persistence [3]. Recent syntheses confirm that mechanical scarification is typically the most effective dormancy-breaking treatment in *Astragalus* and related taxa, reflecting the dominant role of seed coat impermeability in regulating germination [4]. Such dormancy mechanisms are often associated with extended seed longevity, although empirical data on the upper limits of seed longevity remain scarce, especially for herbaceous legumes.

Herbarium collections represent a unique but underutilized resource for studying exceptional seed longevity under long-term dry storage. Several studies have demonstrated that seeds stored under dry conditions in biological collections can retain the ability to germinate for decades or even centuries, although germination capacity is generally expected to decline over time [5,6]. Samples were obtained from herbarium collections of *Bupleurum tenuissimum* (*Apiaceae*), a species currently extinct within the Belgian flora [5]. Researchers successfully germinated seeds that were approximately 144 years old; however, the resulting seedlings were not cultivated to maturity. Nevertheless, the longest officially corroborated germination record within the *Fabaceae* family pertains to *Cassia multijuga* (now classified as *Senna multijuga*), derived from a 158-year-old herbarium specimen [6]. Importantly, recent work highlights that herbarium-derived seeds may serve as a last resort for conservation and restoration, enabling the resurrection of extinct or critically endangered populations [7,8]. However, the extent to which germination capacity is maintained over long periods and the shape of its decline through time remain poorly resolved and are likely species-specific.

*Astragalus contortuplicatus* L. is a rare annual species of periodically flooded habitats in Eurasia, characterized by sporadic occurrence and strong temporal variability in population size. Its annual life cycle, sporadic occurrence, and occupancy of periodically flooded habitats suggest that persistent seed banks may play an important role in its long-term population persistence. In a previous study, we demonstrated [9] that seeds of this species stored in herbarium collections can retain the ability to germinate for exceptionally long periods, with successful germination recorded from seeds up to 131 years old. Moreover, a significant negative relationship between seed age and germination was detected using linear regression, and the theoretical maximum longevity was estimated at 309 years.

Despite these findings, several key questions remain unresolved. First, the long-term stability of seed germination capacity under continued dry storage is unknown, as previous analyses were based on a single time point. Second, the assumption of a linear decline in germination capacity has not been critically tested using repeated observations or non-linear modelling approaches. Third, it is unclear to what extent variability among herbarium specimens reflects stochastic processes, dormancy dynamics, or differences in storage conditions.

In this study, we revisit the long-term germination capacity of *Astragalus contortuplicatus* seeds by repeating germination experiments after an additional 11.5–12 years of storage, using the same methodological protocol as in the original study. By comparing paired observations from the same herbarium specimens and analysing the combined dataset across sampling years, we address the following questions:

(1) Has germination capacity declined over time within the same herbarium specimens?

(2) How does seed age influence germination probability when considering both original and repeated measurements?

(3) Is the relationship between seed age and germination capacity adequately described by a linear model, or does it follow a non-linear pattern?

## 2. Materials and Methods

### 2.1. Seed Material and Study Design

Seed germination capacity of *Astragalus contortuplicatus* was assessed using herbarium specimens originating from Hungarian and neighbouring populations, following the same approach as described previously. In the original study, seeds were extracted from herbarium sheets collected between 1835 and 2013, and germination tests were conducted in 2014. In the present study, a subset of these specimens was re-examined after an additional 11.5–12 years of dry storage (in 2026), provided that sufficient seeds remained available.

For the paired comparison, we included all herbarium specimens for which germination tests were performed both in 2014 and 2026. For the analysis of age-dependent germination capacity, we used the complete dataset comprising all specimens tested in 2014 and/or 2026. Specimens for which no seeds were available in 2026 were included only with their 2014 data.

Seed age was defined as the time elapsed between specimen collection and germination testing. For specimens with uncertain collection dates (e.g., “before 1849”), exact age values were not assigned, and these samples were excluded from analyses requiring continuous age variables.

Seeds used in the present study were originally collected from herbarium specimens in 2014. In several cases, a substantial number of seeds was available, and only a fraction of these was used for the initial germination experiments. The remaining seeds were stored in paper envelopes under ambient laboratory conditions in the Herbarium of the University of Debrecen (DE). For the 2026 experiments, a subset of the remaining seeds was used (typically 5–25 seeds per specimen), while the rest were retained under the same storage conditions to allow for potential future re-testing.

### 2.2. Germination Experiments

Germination tests were carried out using the same protocol as in the 2015 study to ensure methodological comparability. Based on preliminary experiments with freshly collected seeds, the most effective dormancy-breaking treatment for *Astragalus contortuplicatus* was identified as mechanical scarification followed by exposure to light during germination. This treatment was therefore applied consistently in both the 2014 and 2026 experiments.

Seeds were scarified mechanically using Bosch red Woodeco P60 120/1305 sandpaper to disrupt the water-impermeable seed coat characteristic of the species. After scarification, seeds were allowed to imbibe water for 24 h. All scarified seeds imbibed water within this period, indicating that the water-impermeable seed coat had been disrupted by the scarification treatment. Seeds were then placed in Petri dishes on 1% agarose gel. Germination tests were conducted at room temperature (22 ± 2 °C) under a 14 h light/10 h dark photoperiod, with a light intensity of approximately 30 μmol m^−2^ s^−1^. No additional viability test was performed on imbibed seeds that failed to germinate; therefore, our experimental results are interpreted in terms of germination capacity rather than seed viability sensu stricto.

Each seed was scored as germinated if the radicle visibly emerged. Germination was monitored over a 30-day period. For each specimen and sampling year, the number of germinated and non-germinated seeds was recorded, and the germination percentage was calculated as the proportion of germinated seeds relative to the total number of seeds tested. Because non-germinated seeds were not subjected to a separate viability test, the term “non-germinated” is used descriptively and should not be interpreted as direct evidence of seed death.

Germinated seedlings were transplanted to soil at the two-cotyledon stage and maintained under regular watering. Plants were grown on a laboratory windowsill, where they received diffuse natural daylight supplemented with artificial illumination for 12 h per day using Videx 8W E27 lamps. Plants were cultivated until maturity to confirm successful seedling establishment and reproductive development.

### 2.3. Statistical Analysis

All statistical analyses were conducted in R v4.5.3 [10], using binomial modelling approaches appropriate for proportional germination data.

To evaluate changes in germination between 2014 and 2026, we analysed the subset of herbarium specimens that were tested in both years. Germination was modelled as a binomial response (number of germinated vs. non-germinated seeds) using a generalized linear model (GLM) with a logit link function. The testing period (2014 vs. 2026) was included as a fixed effect, while specimen identity was incorporated as a blocking factor to account for the paired structure of the data: germination ~ period + specimen (in R syntax).

Effect sizes were expressed as odds ratios (ORs) with 95% confidence intervals.

To assess the effect of seed age on germination probability across the full dataset, we fitted binomial models using both standard GLM and generalized estimating equations (GEE). In the GEE framework, specimen identity was treated as a clustering variable to account for repeated observations of the same herbarium specimens across different testing years, assuming an exchangeable correlation structure.

The baseline model included seed age (years) as a continuous predictor and testing period as a categorical covariate: germination ~ age + period.

To evaluate potential non-linearity in the age–germination relationship, we compared three alternative model formulations:

- Linear age effect;
- Quadratic age effect (age + age^2^);
- Natural spline model (ns(age, df = 3)).

Model performance was compared using Akaike’s Information Criterion (AIC), and likelihood ratio tests were used where appropriate. The best-supported model was used for visualisation and inference.

Samples lacking precise age estimates (e.g., “>165 years”) were excluded from analyses requiring continuous age variables but were retained in descriptive summaries. Observations with missing germination data (e.g., due to a lack of seeds in 2026) were excluded from model fitting but are included in the full dataset (Table S1).

The relationship between seed age and germination probability was visualised using predictions from the fitted binomial model. Predicted germination probabilities were plotted as a function of seed age, with separate curves for each testing period. Point sizes were scaled according to the number of seeds tested per observation, and 95% confidence intervals were displayed as shaded ribbons.

## 3. Results

**Figure 1:**
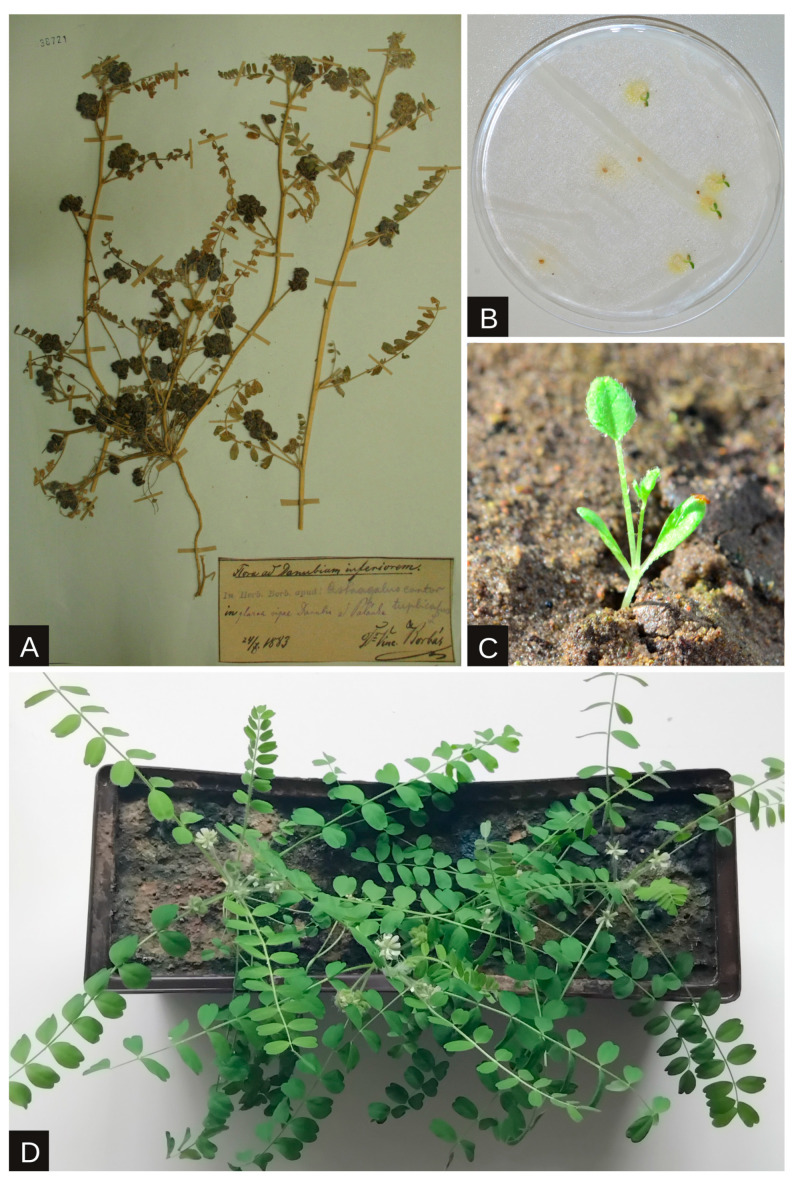
(**A**) Herbarium specimen of *Astragalus contortuplicatus* collected by Borbás in 1883; (**B**) germinating seeds from this material in 2026 after approximately 142.5 years of dry storage; (**C**) seedling; (**D**) plant raised from the germinated seed.

The germination capacity of seven herbarium specimens previously tested in 2014 was reassessed in 2026. A consistent qualitative pattern emerged: all samples that had germinated in the earlier experiment did so again, whereas the single specimen that had previously failed to germinate (collected in 1908) again did not germinate. The resulting seedlings developed into healthy individuals that reached maturity, flowered, and set seed (Figure 1). Voucher specimens of these cultivated plants were deposited in the herbarium of Debrecen University (DE).

Among the herbarium specimens tested in both 2014 and 2026, germination responses varied markedly, with some samples showing pronounced declines (Table 1). A paired binomial analysis revealed a significant overall reduction in germination probability over the 11.5–12-year interval. The odds of germination in 2026 were significantly lower than in 2014 (odds ratio [OR] = 0.443, 95% CI: 0.238–0.824, *p* = 0.010), corresponding to an approximately 55.7% decrease in germination odds across the re-tested specimens.

**Figure 2:**
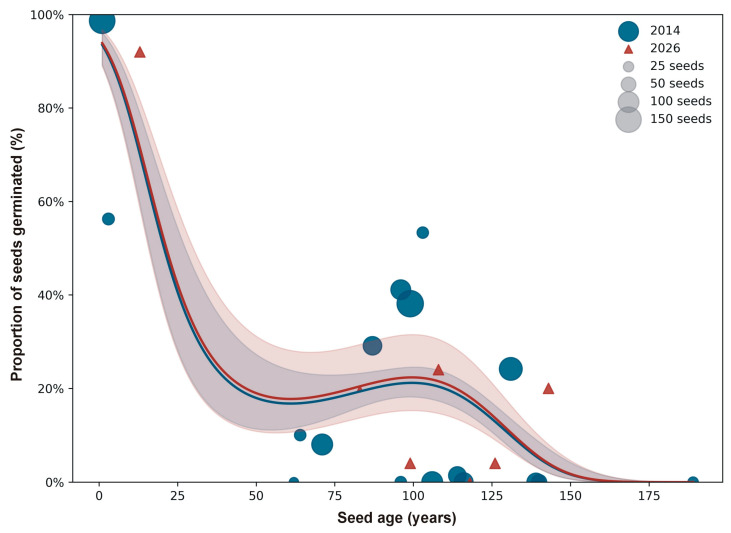
Relationship between seed age and the proportion of seeds germinated in *Astragalus contortuplicatus*. Points represent observed germination proportions for individual herbarium specimens, scaled by the number of seeds tested. Lines show fitted spline-based binomial model predictions, with shaded areas indicating 95% confidence intervals. Separate curves are shown for the two sampling years (2014 in blue and 2026 in red).

To assess the overall relationship between seed age and germination across the combined dataset, we fitted a logistic marginal model using generalized estimating equations, with specimen identity treated as the clustering variable to account for repeated observations of the same herbarium sheets. Seed age showed a significant negative effect on germination (OR per year = 0.973, 95% CI: 0.958–0.988, *p* < 0.001), demonstrating that germinability declines with increasing storage duration. The fitted spline model revealed a clearly non-linear decline in germination probability, with high variability among specimens of similar age (Figure 2). The full dataset used for modelling, including all germination tests conducted in 2014 and 2026, is provided in Table S1. In contrast, the additional effect of testing year (2026 vs. 2014), after accounting for seed age, was not supported (OR = 0.757, 95% CI: 0.431–1.329, *p* = 0.332).

Because the original 2015 study used a linear regression framework, we further evaluated whether the age–germination relationship was adequately described by a simple linear term. Model comparison showed that non-linear age-response functions provided a better fit than a strictly linear logistic model. Specifically, the quadratic model improved model fit relative to the linear model, and a spline-based logistic model performed best (AIC_linear = 412.39, AIC_quadratic = 398.45, AIC_spline = 357.72). This indicates that the decline in germination probability with seed age is non-linear, with substantial between-sample heterogeneity superimposed on the overall downward trend.

## 4. Discussion

The present re-assessment confirms the central conclusion of the 2015 study [9], namely that seeds of *Astragalus contortuplicatus* retained under herbarium conditions can retain the ability to germinate for exceptionally long periods. The new experiments demonstrate that this remarkable longevity persists even after a further 11.5–12 years of dry storage, as several historically collected samples still germinated and produced seedlings that developed into healthy plants in 2026 (Figure 1). Historical collections have repeatedly provided evidence for exceptional seed longevity beyond the range typically observed under experimental conditions [5,11,12,13]. At the same time, the paired comparisons demonstrated that germination capacity was not temporally constant across all re-tested specimens: germination rates decreased in four cases and increased in three between 2014 and 2026, although the magnitude and direction of these changes should be interpreted cautiously because of the limited number of seeds available for several 2026 tests. It should be noted, however, that the present study did not directly examine the soil seed banks of *A. contortuplicatus*. Therefore, our results should be interpreted as indirect evidence that long-lived dormant seeds may contribute to population persistence, rather than as direct confirmation of persistent soil seed banks in natural populations.

The observed temporal decline in germination capacity is biologically plausible and may reflect progressive deterioration during prolonged dry storage. Because all scarified seeds imbibed water, the lack of imbibition is unlikely to explain the reduced germination observed in some samples. Nevertheless, because imbibed but non-germinated seeds were not subjected to an independent viability test, we cannot determine whether failure to germinate reflected loss of viability or other causes of non-germination under the experimental conditions. The pattern is not strictly uniform among specimens. Such variability is consistent with previous findings that seed longevity is strongly species- and context-dependent [14]. Some samples exhibited pronounced reductions in germination, whereas others remained relatively stable, and in a few cases, apparent increases were observed. Given the small number of seeds available for several 2026 tests, such irregularities are most parsimoniously explained by sampling variance combined with heterogeneity in dormancy status and long-term storage history. Thus, the new dataset supports an overall decline in germination capacity but also underscores that stochasticity and specimen-specific storage conditions remain major determinants of observed germination. An important implication of the present analysis is methodological. Although the original paper fitted a linear regression and derived a theoretical maximum seed longevity of 309 years, the expanded dataset now indicates that such linear extrapolation is unlikely to provide a robust estimate of true longevity. The relationship between seed age and germination probability is better described by a non-linear binomial model, as shown by the substantially improved fit of spline-based regressions. This result is in line with recent conceptual work emphasizing that decline in seed germination capacity is rarely linear and depends strongly on species-specific traits and storage conditions [15]. This is unsurprising from a biological perspective because seed ageing is rarely expected to proceed as a simple linear process on the probability scale.

Accordingly, we suggest that future analyses should avoid interpreting herbarium seed longevity in terms of a single extrapolated maximum age. Notably, seeds from the oldest specimen (collected in 1883) were still able to germinate after approximately 142.5 years, producing healthy plants (Figure 1). A more defensible formulation would emphasize that seeds of *Astragalus contortuplicatus* show extraordinary longevity under dry herbarium storage, with empirically demonstrated germination extending beyond 140 years in the updated dataset, while the probability of germination declines significantly with age and is best modelled using non-linear binomial approaches. In this framework, herbarium collections remain highly valuable for conservation and restoration purposes, but predictions of absolute longevity should be expressed cautiously and with explicit consideration of model uncertainty.

## Figures and Tables

**Table 1:** Germination results of herbarium specimens of *Astragalus contortuplicatus* tested in 2014 and re-tested in 2026. For each specimen, the year of collection, seed age at testing, number of germinated and tested seeds, and germination percentage are provided. Data for 2014 are from our previous study [9], whereas data for 2026 are based on the present study. Abbreviations: BP = Herbarium Carpato Pannonicum of Botanical Department of Hungarian Natural History Museum (Budapest), DE = Herbarium of Botanical Department of University of Debrecen (Debrecen).

Year of Collection (Age in 2026)	Country: Locality & Collector	Herbarium Source	2014		2026		Δ Germination (%)
			Number of Seeds Germinated (Tested)	Germination Percentage (%)	Number of Seeds Germinated (Tested)	Germination Percentage (%)	
1883 (143)	Serbia: Bačka Palanka (‘Palánka’), Borbás V.	BP	29 (120)	24.2	5 (25)	20.0	−4.2
1900 (126)	Hungary: Tiszaalpár, Wagner J.	BP	1 (71)	1.4	1 (25)	4.0	+2.6
1908 (118)	Hungary: Szeged, Lányi B.	BP	0 (100)	–	0 (15)	–	–
1918 (108)	Serbia: Novi Bečej (‘Törökbecse’), Boros Á.	BP	37 (90)	41.1	6 (25)	24.0	−17.1
1927 (99)	Hungary: Kunszentmárton, Tamássy G.	BP	23 (79)	29.1	1 (25)	4.0	−25.1
1943 (83)	Hungary: Szeged, Timár L.	BP	8 (100)	8.0	1 (5)	20.0	+12.0
2013 (13)	Hungary: Tiszaroff, Lovas-Kiss Á. & Molnár V. A. (cultivated)	DE	148 (150)	98.7	23 (25)	92.0	−6.7

## Data Availability

All data generated and analysed during the present study are included in this article (Table 1) and its Supplementary Materials (Table S1).

## Supplementary Materials

The following supporting information can be downloaded at https://www.mdpi.com/article/10.3390/plants15142156/s1; Table S1: Age and origin of herbarium samples used in germination tests and the results of the tests. 2014 data from Molnár V. (2015) [9], 2026 data based on recent experiments.
