# Supplementary material for: Remarkable Longevity of Herbarium-Derived Seeds of the Rare and Threatened Annual Legume Astragalus contortuplicatus L.: Germination After More Than 142 Years of Dry Storage
Source: Plants (Basel). 2026 Jul 13;15(14):2156. doi: 10.3390/plants15142156 (PMC13414612; doi:10.3390/plants15142156)
Supplement: Supplementary file 1 [file plants-15-02156-s001.zip › plants-4397999-supplementary.pdf]

Supplementary file

Table S1. Age and origin of herbarium samples used in germination tests and the results of the tests. 2014 data from Molnár V. (2015), 2026 data based on recent experiments.

	Year of collection (Age in years)	Country: Locality & Collector	Herbarium source	2014: Age in years	2014: Germination percentage (%)	2026: Age in years	2026: Germination percentage (%)
1	1835 (189)	Romania: Banat, Rochel A.	BP	189	0	201	NA
2	before 1849 (>165)	Hungary: Tiszabő, Sadler J.	SAMU	>165	0	>177	NA
3	before 1849 (>165)	Hungary: Tiszabő, Sadler J.	BP	>165	0	>177	NA
4	1874 (140)	Hungary: Szarvas, Koren I.	BP	140	0	152	NA
5	1875 (139)	Hungary: Békés county, Koren I.	BP	139	0	151	NA
6	1883 (131)	Serbia: Bačka Palanka ('Palánka'), Borbás V.	BP	131	24.2	143	20.0
7	1898 (116)	Serbia: Budva ('Torontál, Beodva'), Thaisz L.	BP	116	0	128	NA
8	1900 (114)	Hungary: Tiszaalpár, Wagner J.	BP	114	1.4	126	4.0
9	1908 (106)	Hungary: Szeged, Lányi B.	BP	106	0	118	0
10	1911 (103)	Serbia: Deliblat, Wagner J.	DE	103	53.3	115	NA
11	1914–1915 (99)	Serbia: Bečej ('Óbecse'), Kovács F.	DE	99	38.1	111	NA
12	1918 (96)	Serbia: Novi Bečej ('Törökbecse'), Boros Á.	BP	96	41.1	108	24.0
13	1918 (96)	Serbia: Novi Bečej ('Törökbecse'), Boros Á.	BP	96	0	108	NA
14	1927 (87)	Hungary: Kunszentmárton, Tamássy G.	BP	87	29.1	99	4.0
15	1943 (71)	Hungary: Szeged, Timár L.	BP	71	8.0	83	20.0
16	1950 (64)	Hungary: Tápé, Timár L.	DE	64	10.0	76	NA
17	1952 (62)	Hungary: Szolnok, Csapody V.	BP	62	0	74	NA
18	2011 (3)	Hungary: Tiszaroff, Lovas-Kiss Á. & Sramkó G.	DE	3	56.3	15	NA
19	2013 (1)	Hungary: Tiszaroff, Lovas-Kiss Á. & Molnár V. A. (cultivated)	DE	1	98.7	13	92.0
